# A cavalpulmonary assist device utilising impedance pumping enhanced by peristaltic effect

**DOI:** 10.1177/03913988241268419

**Published:** 2024-09-02

**Authors:** Arthur P. Burns-Cox, Lian Gan, Ashraf W. Khir

**Affiliations:** Department of Engineering, Durham University, Durham, UK

**Keywords:** Paediatric VAD, pulsatile, non-invasive to blood, valveless, impedance, peristalsis

## Abstract

**Background:**

Fontan procedure, the standard surgical palliation to treat children with single ventricular defects, causes systemic complications over years due to lack of pumping at cavopulmonary junction. A device developed specifically for cavopulmonary support is thus considered, while current commercial ventricular assist devices (VAD) induce high shear rates to blood, and have issues with paediatric suitability.

**Aim:**

To demonstrate the feasibility of a small, valveless, non-invasive to blood and pulsatile rotary pump, which integrates impedance and peristaltic effects.

**Methods:**

A prototype pump was designed and fabricated in-house without any effort to optimise its specification. It was then tested in vitro, in terms of effect of pumping frequency, background pressure differences and pump size on output performance.

**Results:**

Net flow rate (NFR) and maximum pressure head delivery are both reasonably linearly dependent on pumping frequency within normal physiological range. Positive linearity is also observed between NFR and the extent of asymmetric pumping. The device regulates NFR in favourable pressure head difference and overcomes significant adverse pressure head difference. Additionally, performance is shown to be insensitive to device size.

**Conclusions:**

The feasibility of the novel rotary pump integrating impedance and peristaltic effects is demonstrated to perform in normal physiological conditions without any optimisation effort. It provides promising results for possible future paediatric cavopulmonary support and warrants further investigation of miniaturisation and possible haemolysis.

## Introduction

Functional single ventricular defects are a group of congenital cardiac malformations characterised by a lack of two well-developed ventricles and not amendable via biventricular repair.^1^ Single ventricular defects are rare (85 per million live births) but of increasing concern, not least because of the high healthcare utilisation of this patient group. Without intervention in early life, survival to adulthood is extremely rare and death in childhood is typical.^2^ Now, the Fontan procedure^3^ and its modifications are the standard surgical palliation to treat children with single ventricular defects, although it was originally proposed just to repair tricuspid atresia.

The principle of the Fontan procedure is to bypass the subpulmonary ventricle by routing the systemic venous return directly to the pulmonary circulation, so that the single ventricle is committed to the systemic circulation and the load on the ventricle can be reduced. This neoportal system is often called Fontan circulation. It introduces significant alteration of the normal cardiovascular haemodynamics, due to the reversal of pressure gradient between caval veins and arteries compared to normal biventricular circulations and this causes long term efficiency declination and ultimately leads to fatal systemic complications, such as hepatic failure.

Systemic complications are largely due to upstream venous congestion and downstream decreased cardiac output. In the absence of a pumping chamber to the pulmonary circulation, the single ventricle, which drives the entire circulation has to pull blood through the lungs using a degree of suction which is not physiological.^4^ As a result, the systemic venous pressure is markedly elevated, as is the hepatic venous pressure.^5^

Therefore the key goal is to reduce systemic venous congestion whilst at the same time lowing pressure in the pulmonary circulation to maintain the pressure gradient. An effective way is to provide subpulmonary assistance, which will permit systemic pressure to be lower than pulmonary pressure and provide sufficient power to overcome pulmonary vascular resistance, the critical contributor to Fontan failure. Nevertheless, all commercial ventricular assist devices (VADs) presently used are primarily designed for systemic arterial support. They are not specifically designed for the widely variable cardiac morphology of Fontan patients. The worldwide experience in supporting the pulmonary circulation in failing Fontan patients is very limited to few successful short-term cases.^6^ This motivates us to develop a novel cavopulmonary assist device to provide the 10 mmHg pressure head delivery^7^ to overcome the bottleneck in the Fontan circulation.

Integrating the principles of impedance and peristaltic pumping, the proposed device will demonstrate in vitro pulsatile (vs continuous flow in most commercial VADs) and unidirectional flow without the need for a one-way valve. It is impeller-free with no blood contact. Most importantly, the device could be synchronised with the single ventricle via the detection of intracardiac electrocardiograms, hence potentially supporting exercise.

### Impedance pumping

This pumping via applied periodic forcing produces controllable and unidirectional flow in pipes without the need of one-way valves.^8^ This phenomenon, first discovered by Liebau in 1955,^9^ utilises periodic compression of a flexible tube at an asymmetrical position to generate unidirectional and pulsatile flow. The flexible tube is connected to a more rigid tube of differing impedance at either end, and the generated flow rate is non-linearly related to the pinching frequency.

**Figure 1.**
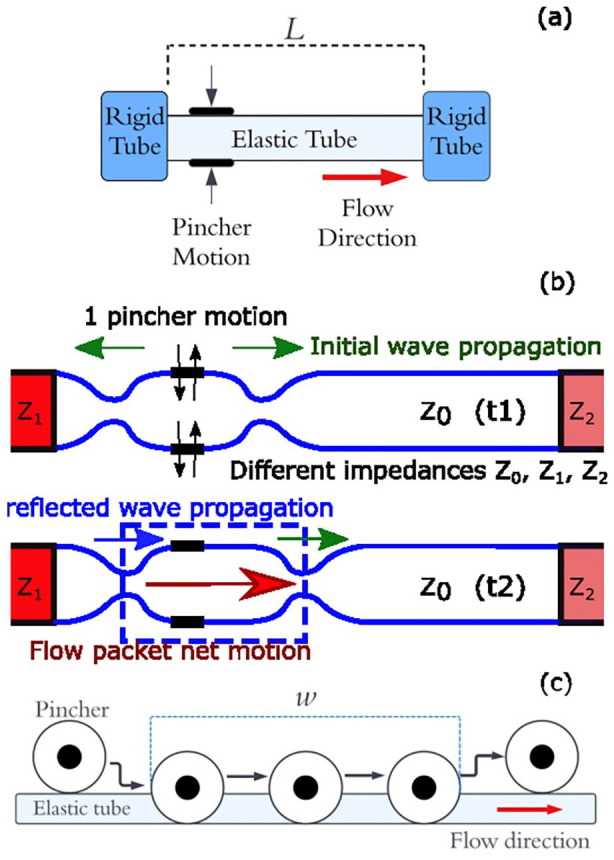
(a) Impedance pumping arrangement. (b) Wave propagation in the pliable elastic tube. (c) Peristaltic pumping mechanism.

The working principle is summarised in Figure 1(a) and (b) which show pressure wave propagation at the start of a single pumping cycle. A single pinching-release motion takes place at an off-central location at time t1 . The pressure waves propagate at wave speed in opposite directions via inertia until reflection occurs at the entrance to rigid tubes due to their higher impedance. At a later time t2 both pressure waves move towards the right hand side of the tube, with the left most wave having reflected due to the off-central pincher location. The pressure waves both move at wave speed to the right hand side of the tube. The main contributor to net flow is the suction effects in the pumping zone towards the outlet of the pipe. Here, the pressure wave from the pincher and the reflected pressure wave from the previous cycle meet and form an area of high pressure, locally expanding the tube and creating an area of volume suction. Two flow waves from either direction then are drawn into this space, with the resulting flow moving in the direction of whichever wave had more energy before collision, which is itself dependant on the frequency of pinching and system parameters.^10,11^

Analysis of the closed loop system was first carried out by Thomann.^12^ They demonstrated valveless pumping through a torus made of two elastic tubes of differing elasticity via pinching of the more pliable tube, and modelling of the system using one-dimensional Navier-Stokes equation. This approach was furthered by Moser,^13^ demonstrating impedance-defined flow with a closed loop and elastic tube. They suggested that impedance and wave effects are the dominating factors in net pumping, rather than fluid inertia. Ottensen^14^ devised a one-dimensional single loop model and compared model performance to qualitative experimental results. Their findings showed a dependence of the net flow rate (NFR) on pinching frequency, location, duty cycle and tube elasticity. The modelling attempt by Borzì and Propst^15^ demonstrated the requirements for a net pressure head from open reservoir pumping. The open loop configuration reduces the effect fluid inertia can have on the pumping mechanism, and suggested that wave mechanics within the flexible tube were likely to be a governing factor in impedance pumping. Hickerson et al.^16^ investigated pincher position, actuation frequency, materials and systemic resistance using both open and closed loop setups. It was determined that locating the pincher at the end of the flexible tube led to greater maximum NFR, and that NFR went through apparent resonant peaks as frequency increased, which changed with pinching locations.

Interest in biomedical applications of impedance pumping has been growing. Pahlevan and Gharib^17^ found that the embryonic human heart functions as a valveless pump due to impedance effects, rather than peristalsis as previously thought. Zislin and Rosenfeld^18^ investigated impedance pumping performance in a multi vessel system and concluded that impedance pumping may be a suitable method for flow control of the cardiovascular system. Davtyan and Sarvazyan^19^ researched the performance of impedance pumping with biologically relevant vessel properties and actuation frequencies and found that impedance pumping could produce similar NFR to similarly sized peristaltic pumps, a remarkable feat due to the lower energy and smaller tube length compressed by the impedance pump. Building on this, Sarvazyan^20^ suggested tissue-based pinching methods with muscle rings, and body sites suitable for biologically implementable Liebau pumps. Recently, Anatol et al.^21^ investigated purely asymmetric valveless pumping with biologically relevant vessel sizes and actuation frequencies. The study concluded that the experimental setup could provide suitable NFR and pressures for paediatric extra-vascular support with a mechanically simple device using only asymmetric pumping and neglecting the need for impedance effect. However, the large size apparatus means the system can be operated at its resonant frequency, whilst on a smaller size the resonant frequency would most likely be too high to suit biologically useful frequencies.

### Peristaltic pumping

Peristaltic pumping is a form of positive displacement pumping achieved by displacing fluid with a roller inside a compliant tube, forcing fluid forwards and creating net suction behind the roller, resulting in a net positive pumping effect,^20^ Figure 1(c). The rollers compress the tube in a similar manner to the impedance pump, but flow direction is controlled by the direction of the rollers motion and the valve behaviour of the rollers, forcing forward flow and restricting backflow.^22^ NFR is therefore directly proportional to forcing frequency, the compressed tube length and tube diameter. Flow reversal is only achievable by reversing the direction of the roller compression and, unlike the impedance pump, resonance effects do not affect flow rate. The simplicity and valveless feature make it a useful pump for low flow rates, alongside low fluid shear rates, associated with blood cell damage, due to lack of direct fluid contact. The peristaltic pump has many applications and is particularly useful for accurate dose delivery due to the precision in volume displacement.^23^ However it must be large in size to support sufficient flow rate and therefore are not well suited to be used alone as a VAD.

## Experimental method

### Pinching mechanism

**Figure 2.**
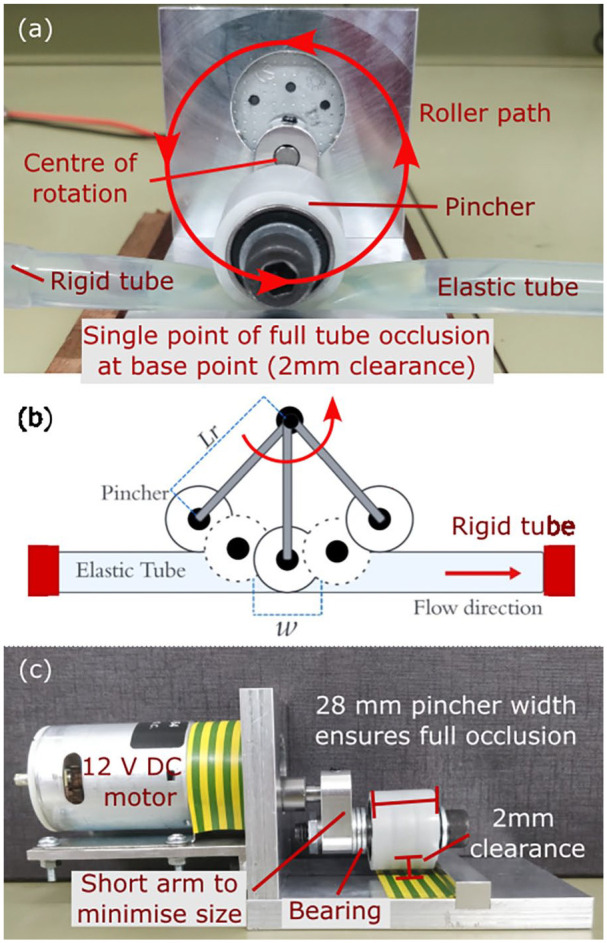
Rotary pumping mechanism: (a) front view, (b) schematics of rotary tube occlusion, and (c) side view. Pincher diameter ϕ=27mm , rotor arm length Lr=22mm .

A prototype of the device is shown in Figure 2. A roller type pincher controlled by a variable speed DC motor was attached to a rigid aluminium frame. The pincher fully occludes the tube at the bottom of its rotation whilst not contacting it at the top. The period of pinching, determined by the arm length Lr , is short compared to the total rotation period, which improves performance as per Moser’s first condition.^13^ The use of the rotary mechanism allows the pincher motion to be small, smooth, quiet and require low power. The DC motor allows precise pumping frequency (f) control, and the roller with bearing minimises resistance due to friction. In this feasibility study, we did not aim to optimise any specification of the device, for example, the DC motor used is an existing off-the-shelf product, which is oversized and overpowered for the current purpose.

Figure 2(b) illustrates the action of the rotary pump, which is an unexplored pinching mechanism for impedance pumping to the best of our knowledge. The extent contributed by the peristaltic effect from the roller ‘pushing’ the fluid alongside impedance effects is investigated. Peristaltic NFR, $Q_{p}$ , can be expressed as^20^:

(1) $$Q_{p}=\left(\pi r_{i}^{2}\right)fW,$$

where $r_{i}$ is the inner radius of the assuming circular elastic tube and W is pincher compression range; see also Figure 1(c), the length of the tube that is fully occluded during a pumping cycle. When compared to a classical peristaltic pump, the compression range is smaller for the current device and so is the peristaltic NFR, $Q_{p}$ , as per equation (1). However, tube pinching at a single occlusion point provides simplicity, robustness and allows easier device miniaturisation.

### Experimental testbed

**Figure 3.**
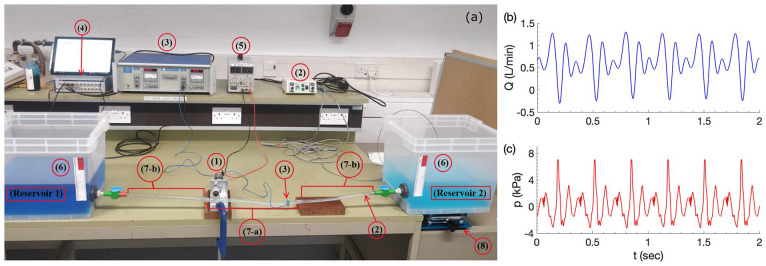
Experimental testbed (a). Typical instantaneous waveforms at pumping frequency 3Hz for flow rate Q (b) and static pressure p (c).

The experimental testbed is displayed in Figure 3. Two plastic water reservoirs, each with base size 300mm×220mm , labelled as (6), are attached at both ends of the open circuit, where water as the testing liquid is coloured by food dye for visualisation purpose. The size of the reservoir was chosen to be large and to minimise water level change (i.e. pressure head change) during each experiment. The open circuit is made from a pliable elastic tube (7-a) and a rigid tube (7-b) at each end; see also Figure 1. The rotary pump, (1), is located within the elastic tube span and powered from a variable voltage power pack, (5). The connection of the elastic tube to the two rigid tubes is via push fit. Properties of the tubes utilised are displayed in Table 1.

**Table 1.** Experimental tube parameters. $r_{i}$ : inner radius, $r_{o}$ : outer radius, E : Young’s modulus.

Tube	$r_{i}$ (mm)	$r_{o}$ (mm)	E (MN/m^2^)
Elastic1	5	6	1.69
Elastic2	4	5	1.40
Rigid1	5.5	6.5	9.38
Rigid2	3.5	4.5	9.99

The off-the-shelf tubing size used is selected purposely to be within physiological ranges, with the internal diameter of the pulmonary vein, for example, commonly within the range of 9–13 mm.^24^ The tubing is slightly smaller than the major cardiac arteries in the adult,^25^ but close to that of the paediatric population with mean proximal aorta diameter only about 10 mm for children between 3 and 8 years old.^26^

### Data collection method

Instantaneous flow rates were measured non-invasively using a Transonic Systems T206 Dual Channel Small Animal Blood Flow Meter with Transonic Flow Probes (3). Pressure was measured by a Millar PCU-2000 Dual Channel Pressure Control Unit with a Mikro-tip catheter pressure transducer (2). A lab stand (8) was used to elevate the reservoir to create background pressure gradients.

Flow rate and pressure waveform data were collected using an ADInstruments PowerLab signal conditioner (4), sampled at 1 kHz, sufficiently high to avoid signal aliasing. NFR was calculated by averaging instantaneous flow for a minimum of 8 s after steady state condition is reached that is, a minimum transient period of of 2 s is allowed before measurement is taken. Figure 3(b) shows typical instantaneous flow and pressure waveforms, where momentary flow reversal and pressure wave reflection can be clearly observed.

## Results

### Effect of pumping frequency

**Figure 4.**
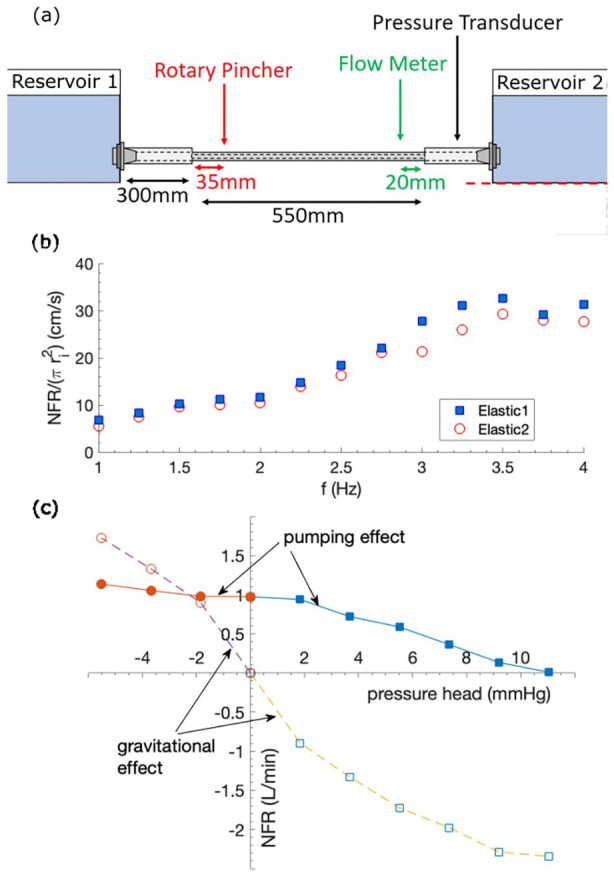
(a) Experimental setup to test the effect of pumping frequency; not to scale. (b) Effect of f on NFR, normalised by the cross-sectional area of the elastic tube $\pi r_{i}^{2}$ . (c) Effect of pressure head on NFR at f=2.5Hz . Filled symbols are for pumping effect and open symbols are solely due to gravitational effect.

Experimental setup to investigate the effect of pumping frequency on NFR is illustrated in Figure 4(a). Note that the rotary pincher is located at an off-central location. In order to examine the effect of tube size, two sets of elastic-rigid tube pairs, as described in Table 1, are tested. Owing to the push fit connection method, the size of the rigid tube needs to vary with the elastic tube size. The results are shown in Figure 4(b), which suggests an approximately linear NFR increment with f , for f<3.5Hz . At higher frequency, f≳3.5Hz , NFR plateaus, suggesting that the reverse flow due to wave interactions from the impedance effect starts to emerge for the particular elastic tube length utilised.

It is also evident from Figure 4(b) that NFR is proportional to the elastic tube cross-sectional area as the data points reflect good degree of collapse over the tested f range. It is for this reason that we will only present results from Elastic1 (L=550mm) and Rigid1 (300mm) , as shown in Figure 4(a), unless otherwise specified.

### Effect of pressure head

The ability for the pump to deliver pressure head, that is, produce flow against an adverse (positive) pressure, is essential for a VAD. That is, pressure head delivery of 110 mmHg for the full systemic support and 10 mmHg for cavopulmonary support; see § 1. To test this, the experimental setup was kept the same as shown in Figure 4(a), but with the additional lab stand, Figure 3(8), to raise Reservoir 2. As the reservoirs had the same water level initially, the adverse pressure head is generated according to ρgH , where H is lab stand height change. The wide base of the reservoirs ensured that instantaneous fluid height change was negligible during pumping. Two valves (not one-way valves) installed at the reservoir exit ensured there was no flow before testing started, maintaining the initial pressure difference condition at the two ends. The pincher operated at the single frequency f=2.5Hz . The behaviour of the pump was also characterised with a favourable (negative) pressure head delivery, that is, with Reservoir 1 raised with the lab stand.

The results are shown in Figure 4(c). Also included for comparison is the gravitational driven NFR, when the pump does not operate.

### Effect of elastic tube length

**Figure 5.**
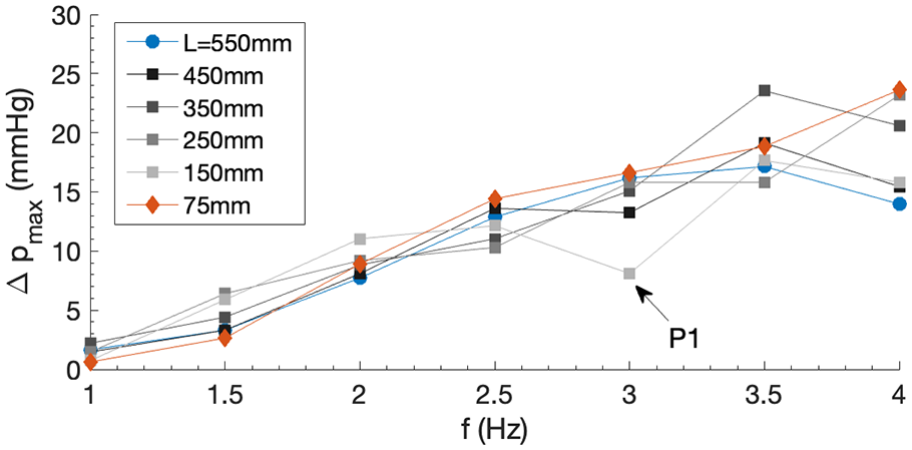
Dependence of maximum pressure head delivery $\Delta p_{max}$ on pinching frequency f .

To explore the feasibility for miniaturisation of the pump system, the effect of length L of the elastic tube on maximum pressure head delivery $\Delta p_{max}$ was investigated by modifying the experimental setup in the way shown in Figure 3(a). L was reduced in step to a minimum of 75mm , given the size of the prototype. To measure $\Delta p_{max}$ , Reservoir 2 was replaced with an upright supported rigid tube open to the atmosphere with a ruler placed alongside. The elastic tube remained straight to avoid wave interference. The initial condition was set such that the level of the water column in the upright tube was at the same level as that in Reservoir 1. When the pump was actuated, the delivered pressure increased the level of the water column to oscillate slightly around an equilibrium point, due to the pulsatile nature of the pumping mechanism, where the water head was measured. A mean height was measured over 10 s. The results are displayed in Figure 5.

### Effect of impedance versus peristalsis

**Figure 6.**
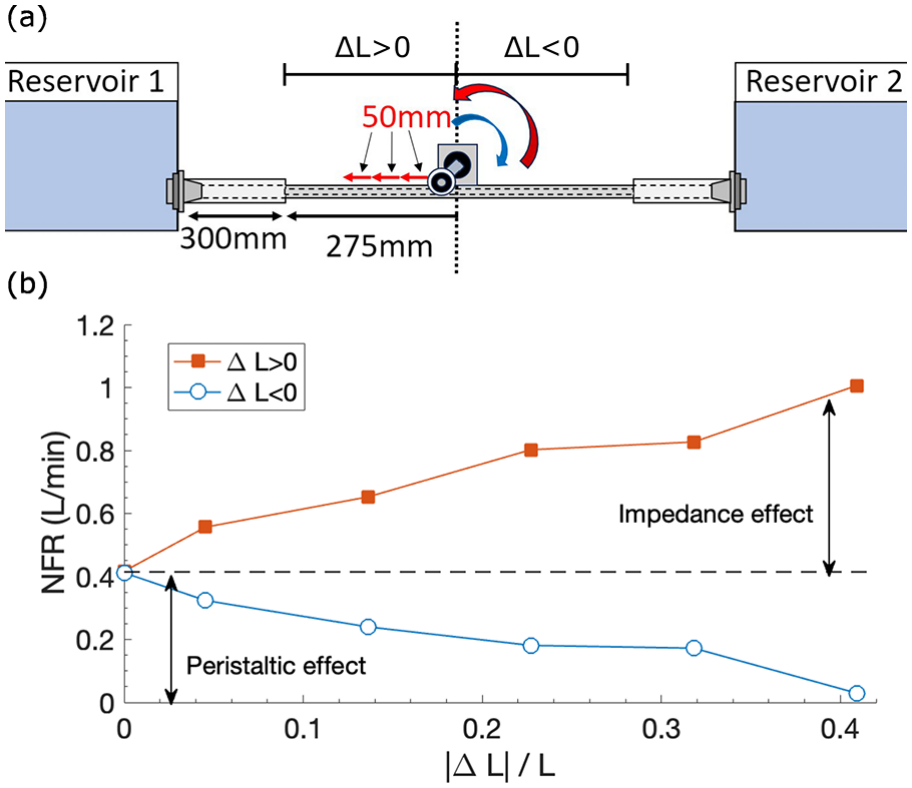
(a) Experimental setup to separate the impedance and peristaltic effect; not to scale. Pincher frequency f=2.5Hz . (b) Dependence of NFR on off-central pincher position ΔL . L=550mm .

As an attempt to separate the effects of impedance and peristalsis on NFR, the pumping position was varied off-centrally, assuming peristaltic pumping does not strongly depend on the pumping position. The experimental design is illustrated in Figure 6(a). At the centre of the elastic tube, ΔL=0 , impedance pumping does not contribute to NFR due to symmetry.^9^ NFR is thus entirely provided by peristaltic effects. As the pincher moves to an off-central position, impedance effects start to contribute, either positively for ΔL>0 or negatively for ΔL<0 . The effect of ±ΔL can also be achieved via changing the motor rotary direction. That is, anti-clockwise rotary motion (the default direction) over the ΔL<0 range is equivalent to clockwise rotary motion over ΔL>0 , for the overall NFR magnitude. Results are presented in Figure 6(b).

## Discussion

In healthy adults, heart beats at 60∼100 beats per minute (BPM), which is equivalent to 1∼1.67Hz at rest, but can increase to 180 BPM (3 Hz) during vigorous exercise. The flow rates required by the heart vary between patients with cardiac malfunction and at changing heart rates. This means that quantification of NFR as a function of actuation frequency f is an important performance indicator of the proposed pumping mechanism.

The linear behaviour of NFR against pumping frequency shown in Figure 4(a) exhibits some similarity to impedance pumping at low frequency,^10,11^ but with improved linearity attributed to the peristaltic pumping effect, equation (1). This improved linear behaviour therefore is arguably better suited for supporting cardiac output compared to pure impedance pumping because of this improved predictability for motor control.

The frequency range investigated, 1≤f≤4Hz , is below the resonant frequencies of the tube, $f_{n}$ , where the flow rate is expected to be maximal.^10^ $f_{n}$ for a compliant tube can be calculated as

(2) $$f_{n}=\frac{c}{2L},$$

where L is elastic tube length (Figure 1(a)) and c is wave travelling speed. c is the rate of propagation of the pressure wave along the compliant tube and is a function of material properties of the tube wall. This can be estimated from the Moens-Korteweg equation for thin-walled flexible tubes, given by:

(3) $$c=\sqrt{\frac{E}{2\rho }\left(\frac{r_{o}-r_{i}}{r_{i}}\right)},$$

where E is Young’s Modulus of the elastic tube (Table 1), $r_{o}$ is elastic tube outer radius and ρ is fluid density. The accuracy of this equation can vary between systems however, where c sometimes might be overestimated significantly.^21^ Equation (3) has been verified in several studies over time,^27^ and further research may be required. For instance, c can also be estimated by^28^

(4) $$c=\sqrt{\frac{1}{\rho D_{s}}},$$

with $D_{s}$ being the distensibility of the elastic tube

(5) $$D_{s}=\frac{1}{\Delta p}\left(\frac{\Delta r_{i}^{2}}{r_{i}^{2}}\right).$$

It represents the fractional change of cross-sectional area upon change of fluid pressure. This equation is a slightly simplified model, and may be of use when E of a tube is difficult to obtain.

For the 2 elastic tubes used in this study, $f_{n}\approx 12\;\;\mathrm{Hz}$ , benchmark estimated from equations (2) and (3), since their E is available. These are considerably higher than physiologically relevant frequencies. However Figure 4 shows that decent performance is achieved at $f\ll f_{n}$ supported by other similar observations.^14,10,19^ Note that NFR, equivalent to 1.6 and 0.8L/min respectively for Elastic1 and Elastic2 at f=3.5 Hz, the range aimed for paediatric circulatory devices^21^ for example, are generated entirely by the pump from resting fluid. With background flow, and if tube material and design are optimised, for example, by reducing stiffness or wall thickness to bring $f_{n}$ down to physiological range, remarkable NFR delivery may be expected.

As expected, Figure 4(c) demonstrates that NFR drops in a reasonably linear manner as the adverse pressure head increases (positive range), but it delivers significant flow against the increasing gravitational effect. For favourable pressure head (negative range), it is interesting to see that the pump is able to regulate NFR in the system, as NFR changing upon pressure head gets flatter compared to that of the pure gravitational effect. This shows that the pump has an active control of system NFR.

The effect of f on $\Delta p_{max}$ shown in Figure 5 echoes its effect on NFR in Figure 4(b), where a reasonably linear relation can be observed, which is considered an advantage over a non-linear behaviour leading to better predictability and hence motor control. Results show that L does not impose a strong effect on the pressure head delivery over the tested range. Data appear more scattered for f≳3.5Hz , close to the top of physiological range. This is likely because impedance has a greater effect at higher frequencies (with different $f_{n}$ for different L , equation (2)). Point P1 is an apparent outlier, dropping to a lower pressure value for L=150 mm at f=3 Hz. This is likely due to wave interactions within the tube at this particular frequency negatively interfering and reducing the pressure delivered by the impedance effect.

Results shown in Figure 6(b) illustrate that at ΔL=0 , NFR≈0.4L/min , which agrees with equation (1) considering partial occlusion effect, Figure 2(b). It is evident that NFR contributed from impedance effects are again reasonably dependent on |ΔL| in a linear way, supported by a previous study.^16^ The effect of the sign of ΔL on NFR is roughly symmetric about NFR≈0.4L/min . The slight asymmetry might be attributed to the pressure wave interference effect induced by the peristaltic pumping, which could be mildly position sensitive.

## Limitation

In this feasibility study with in vitro tests, majority of the components used to build the device are off-the-shelf and of low cost. No special effort has been made to optimise any of its specifications, for example, elastic material property or design of motor pumping programmes. Future works include miniaturisation of the whole device and identification of optimal operational conditions, from which greater potential may be expected.

## Conclusion

This study demonstrates a ‘first version’ prototype of a pump, which integrates positive characteristics of impedance and peristaltic effect, and compromises weaknesses of the two. It is non-invasive (no contact with blood), pulsatile, valveless and potentially can be made of small size, features that are not commonly seen in commercial VADs available in the market. Net flow rate and maximum pressure delivery are both reasonably linearly dependent on pumping frequency within normal physiological range. Positive linearity is also observed between net flow rate and the extent of off-central pumping position. It regulates flow rate in favourable pressure head against gravitational effect and linearly overcomes adverse pressure head. Additionally, the length of the elastic tube is shown to have insensitive impact on the pump performance. These features are potentially advantageous for control strategy design and miniaturisation of a cavopulmonary supporting device, which are left for the future study.
